# Tétraplégie révélatrice d'une méningomyélite grave à pneumocoque: à propos d'un cas et revue de littérature

**DOI:** 10.11604/pamj.2015.20.155.6074

**Published:** 2015-02-18

**Authors:** Tahir Nebhani, Hicham Bakkali, Lahcen Belyamani

**Affiliations:** 1 Service d'Accueil des Urgences Médico-chirurgicales de l'Hôpital Militaire d'Instruction Mohammed V, Rabat, Maroc

**Keywords:** Tétraplégie, méningomyélite, streptocoque pneumoniae, Tetraplegia, meningomyelitis, streptococcus pneumoniae

## Abstract

L'atteinte médullaire est une complication rare des méningites à streptocoque pneumoniae. notre observation clinique décrit le cas d'une Jeune femme immunocompétente qui s'est présentée au service d'accueil des urgences pour tétraplégie dont les investigations ont mis en évidence une méningite à pneumocoque. Le traitement était basé sur l'antibiothérapie plus une corticothérapie concomitante. L’évolution était marquée par la persistance des séquelles neurologiques.

## Introduction

La méningite à pneumocoque représente la méningite bactérienne communautaire la plus sévère. Elle s'accompagne souvent de diverses complications neurologiques. Cependant l'atteinte médullaire est une complication extrêmement rare [1]. Nous présentons le cas d'une jeune femme qui a développée une tétraplégie au décours d'une méningite aiguë à pneumocoque.

## Patient et observation

**Figure 1:**
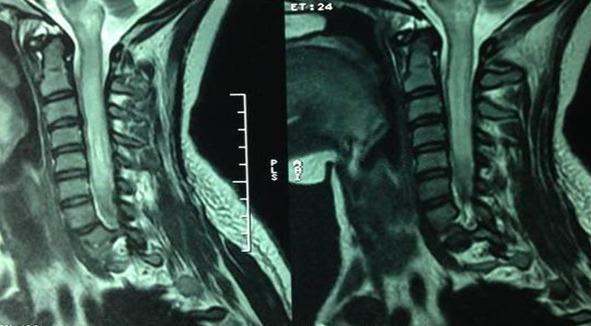
IRM hypersignal T2 étendu sur toute la moelle cervicale qui est gonflé arrivant à la jonction cervico-dorsale

Une femme de 24 ans a été admise au service des urgences, pour un syndrome méningé associé à des signes de focalisations. Elle avait comme antécédent un double remplacement valvulaire mitro-aortique pour lequel elle a été mise sous acénocoumarol à raison d'un comprimé et quart par jour. L'examen clinique trouvait une patiente consciente, apyrétique, avec une raideur de la nuque, un ptosis et une hémiparésie gauches. Devant ce tableau clinique on a pensé à une hémorragie méningée. Le scanner cérébral sans injection était normal. l'International Normalized Ratio (INR) était à 3. L'hémogramme a montré une hyperleucocytose à 12000 cellules par millimètre cube. la protéine c réactive (CRP) était légèrement élevée à 10,7 mg/l. Le tableau clinique 12 heures plus tard s'est complété par l'apparition d'une fièvre à 38,8c associée à une paraplégie flasque, une monoplégie brachiale gauche et un globe vésical. Une tachycardie brutale à 120 battement par minute à QRS fins et une desaturation à 84% à l'air libre nécessitant l'administration d'une dose de charge de 5mg/kg de cordarone et une oxygénothérapie à un débit de 8l/min. L'imagerie par résonnance magnétique (IRM) cérébrale et cervicale a montré une prise de contraste méningée au niveau de la base du crane et un hypersignal T2 de la moelle cervicale évoquant une méningite avec myélite infectieuse (Figure 1). La ponction lombaire (PL) a ramené un liquide céphalorachidien (LCR) jaune clair, avec hypercellularité à 6400 par millimètre cube à prédominance PNN (60%). la coloration de gram a trouvé des cocci gram positif en diplocoque faisant évoquer un *Streptococcus pneumoniae*. Une hyperproteinorrachie à 2,26 g/l et une hypoglycorrachie à 0,52 g/l. La méningite à pneumocoque compliquée d'une myélite a été retenue. Une corticothérapie intraveineuse à base de dexamethasone à raison de 10mg/6h a été administrée suivie par la ceftriaxone (100mg/kg/j) en deux perfusions. La patiente a été transférée en réanimation pour complément de prise en charge. Une échocardiographie transœsophagienne a été prévue pour éliminer une endocardite infectieuse.

## Discussion

Les complications neurologiques de la méningite à pneumocoque fréquemment rencontrées sont représentées par l'atteinte cérébrovasculaire, l’œdème cérébral, l'hydrocéphalie ou l'atteinte auditive [2, 3]. Le dysfonctionnement de la moelle épinière est une complication très rare, trente deux cas ont été décrits au cours des 40 dernières années dont la grande majorité était des enfants [4, 5]. Le signe clinique initial le plus fréquent est la paraplégie ou la tétraplégie [6]. Notre patiente avait présenté initialement une hémiparésie gauche associée à un ptosis et ultérieurement la symptomatologie médullaire est devenue évidente par l’ apparition d'une paraplégie flasque associée à une monoplégie brachiale gauche voir une tétraplégie et une rétention aigue d'urine. Sur le plan physiopathologique l'atteinte médullaire peut être expliquée par trois mécanismes incluant l'ischémie due à une vascularite, la toxicité directe du pneumocoque sur la moelle et l'atteinte auto-immune [7, 8]. La myélite cervicale est plus fréquente chez les nourrissons et les enfants contrairement à la localisation chez notre patiente [9]. L'IRM confirme le diagnostique de myélite.réalisée en urgence, elle permet d’éliminer une compression extramédullaire et met en évidence un hypersignal intramédullaire contigües en T2 étendu de la région cervicale à la région lombaire. Chez notre patiente l'IRM médullaire a montrée un hypersignal T2 étendu sur toute la moelle cervicale qui est gonflée arrivant à la jonction cervicodorsale [7]. Le pneumocoque a été isolé au niveau du LCR. Le pronostic est défavorable quand il s'agit d'une myélite. Des complications hemodynamiques et respiratoires peuvent survenir ainsi que la persistance de séquelles neurologiques [7]. Notre patiente a présentée des tachycardies paroxystiques ainsi que des troubles ventilatoires. Elle avait gardée des troubles sphinctériens.

## Conclusion

La méningite à pneumocoque peut se compliquer d'une myélite. L'examen neurologique répété chez tout malade atteint de méningite doit être la règle. L'IRM médullaire réalisée en urgence est l'examen de choix pour confirmer la myélopathie. La corticothérapie associée à l'antibiothérapie semble justifiées en tenant compte du mécanisme physiopathologique auto-immune probable. Le pronostic est amélioré par la précocité de la prise en charge.

## References

[1] Klaudija Viskovic, Matej Mustapic, Marko Kutlesa, Dragan Lepur (2014). Acute Pneumococcal Myelitis in an Adult Patient. J Glob Infect Dis.

[2] Pfister HW, Feiden W, Einhaupl KM (1993). Spectrum of complications during bacterial meningitis in adults: results of a prospective clinical study. Arch Neurol..

[3] Tunkel AR, Scheld WM (1995). Acute bacterial meningitis. Lancet..

[4] Kikuchi M, Nagao K, Muraosa Y, Ohnuma S, Hoshino H (1999). Cauda equina syndrome complicating pneumococcal meningitis. Pediatr Neurol..

[5] Moffett KS, Berkowitz FE (1997). Quadriplegia complicating Escherichia coli meningitis in a newborn infant: Case report and review of 22 cases of spinal cord dysfunction in patients with acute bacterial meningitis. Clin Infect Dis..

[6] Sodqi M, Marih L, Himmich H (2008). Myélite grave compliquant une méningite à pneumocoque: à propos d'un cas et revue de la littérature. Médecine et maladies infectieuses.

[7] Kastenbauer S, Winkler F, Fesl G, Schiel X, Ostermann H, Yousry TA (2001). Acute severe spinal cord dysfunction in bacterial meningitis in adults: MRI findings suggest extensive myelitis. Arch Neurol..

[8] Khan J, Altafullah I, Ishaq M (1990). Spinal cord dysfunction complicating menin-gococcal meningitis. Postgrad Med J..

[9] Seay AR (1984). Spinal cord dysfunction complicating meningitis. Arch Neuro..

